# Effects of POPG and Cholesterol on Freeze–Thawing Mediated Content Exchange and Transcription in Giant Unilamellar Vesicles

**DOI:** 10.1002/cbic.70594

**Published:** 2026-11-05

**Authors:** Tsubasa Yamashina, Masaya Oki, Gakushi Tsuji

**Affiliations:** ^1^ Department of Applied Chemistry and Biotechnology Graduate School of Engineering University of Fukui Fukui‐shi Fukui Japan; ^2^ Life Science Innovation Center University of Fukui Fukui‐shi Fukui Japan

**Keywords:** artificial cell, cholesterol, flow cytometry, freezing–thawing, liposome fusion

## Abstract

Freeze–thaw (F/T)‐induced content mixing between artificial cells enables efficient exchange of encapsulated components. However, F/T treatment is often accompanied by vesicle rupture and content loss, and the effects of membrane composition on fusion and reaction efficiency remain unclear. Here, we investigated how cholesterol and the negatively charged phospholipid 1‐palmitoyl‐2‐oleoyl‐*sn*‐glycero‐3‐phospho‐(1′‐*rac*‐glycerol) (POPG) influence giant unilamellar vesicle (GUV) stability, content mixing, and transcriptional activity after F/T treatment. Cholesterol‐containing GUVs exhibited increased vesicle loss and reduced content mixing after F/T treatment. In contrast, POPG markedly improved GUV recovery and suppressed vesicle loss, even in the presence of cholesterol. Membranes containing 20 mol% POPG and 20 mol% cholesterol maintained high cargo retention and efficient content mixing. When DNA‐containing liposomes were fused with transcription‐reagent‐containing liposomes through F/T treatment, this membrane composition also yielded the highest transcriptional activity. Notably, although the combination of POPG and cholesterol inhibited transcription in intact GUVs, it showed the highest transcriptional activity following F/T‐mediated content mixing. These findings demonstrate that the effects of membrane composition on encapsulated biochemical reactions depend strongly on whether the reaction components are initially coencapsulated or brought together through F/T‐mediated content exchange.

## Graphical abstract

Phospholipid 1‐palmitoyl‐2‐oleoyl‐*sn*‐glycero‐3‐phospho‐(1′‐*rac*‐glycerol)/cholesterol membranes suppress transcription in intact giant unilamellar vesicles but show the highest RNA synthesis following freeze–thaw (F/T)‐mediated content mixing. These findings highlight the context‐dependent effects of membrane composition on biochemical activity following F/T‐mediated content exchange.

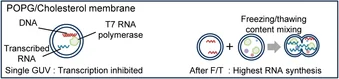

## 1 Introduction

Compartmentalization enables biochemical reactions to proceed efficiently and provides a physical basis for natural selection in primitive life [1, 2]. Modern cells achieve compartmentalization using phospholipid membranes that separate the intracellular and extracellular environments. A wide range of biochemical reactions have been reconstituted within lipid bilayer compartments such as liposomes, including cell‐free protein synthesis [3] and RNA or DNA self‐replication [4, 5]. Cell‐sized giant unilamellar vesicles (GUVs) are widely used as compartments for artificial cell systems [6]. Their cell‐sized characteristics also enable direct observation and quantitative analysis at the single‐vesicle level [7, 8]. However, because most biomacromolecules cannot permeate phospholipid membranes, reactions within GUVs are easily halted once the initial substrates are consumed. Furthermore, genetic materials do not spontaneously spread across individual membrane compartments, which limits recursive self‐reproduction in primitive cell‐like systems. To address these challenges, several studies have developed methods for supplying macromolecules to GUVs via liposome–liposome fusion. There have been developed several fusion methods, including peptide‐mediated [9, 10], DNA‐mediated [11, 12], electrofusion [13, 14], and chemically induced fusion approaches [15, 16, 17, 18]. Among these approaches, freeze–thaw (F/T)‐induced fusion is particularly attractive because cycles of freezing and thawing would have occurred frequently on the prebiotic Earth and thus provide a plausible mechanism for primitive recursive processes [19, 20, 21].

F/T treatment has long been used to encapsulate solutes in small unilamellar vesicles [22] and has recently been applied to repeatedly supply substrates required for RNA replication inside cell‐sized liposomes [19]. Previous work has shown that the freezing temperature strongly affects membrane behavior during F/T [19, 20]. Rapid freezing using liquid nitrogen efficiently mixes both the internal solution and membrane lipids but often causes compartment rupture. We recently demonstrated that the lipid composition of GUVs markedly alters their F/T stability. In particular, incorporation of the negatively charged phospholipid 1‐palmitoyl‐2‐oleoyl‐*sn*‐glycero‐3‐phospho‐(1′‐*rac*‐glycerol) (POPG) reduces vesicle rupture and improves compartment recovery after F/T treatment [23, 24]. These findings indicate that membrane composition is a key determinant of membrane reconstitution during F/T‐induced fusion.

Cholesterol is a major constituent of eukaryotic plasma membranes and is known to modulate membrane organization, permeability, and mechanical properties [25, 26, 27]. Owing to these effects, cholesterol is frequently incorporated into artificial membranes to improve membrane stability and functionality [28, 29, 30, 31]. In addition to their effects on membrane stability and permeability, both cholesterol and charged phospholipids have been reported to influence biochemical reactions within liposomal compartments [24, 32]. These observations suggest that membrane compositions favorable for compartment stability are not necessarily optimal for encapsulated biochemical activity. However, how membrane composition simultaneously affects fusion efficiency and transcriptional reactions remains poorly understood.

In this study, we investigated how cholesterol influences F/T‐induced GUV fusion containing the negatively charged phospholipid POPG. We systematically analyzed GUV retention, inner content mixing, and transcriptional activity in membrane compositions differing in POPG and cholesterol content with or without F/T treatment. Furthermore, we examined how these membrane properties affect RNA synthesis following fusion between DNA‐containing and nutrient‐containing liposomes. Through these analyses, we sought to determine how membrane composition affects compartment recovery and content mixing during F/T, as well as biochemical activity following F/T, and to identify membrane compositions that support these different functions in artificial cell systems.

## 2 Results and Discussion

### 2.1 Fusion Between Liposomes Composed of POPC and Cholesterol by the F/T Method

To examine whether cholesterol influences GUV rupture and inner content mixing during F/T treatment, we prepared GUVs composed of 1‐palmitoyl‐2‐oleoyl‐*sn*‐glycero‐3‐phosphocholine (POPC) containing 0, 20, or 40 mol% cholesterol using the inverted emulsion transfer method. Two types of GUVs were prepared: one encapsulating TA488 and the other encapsulating TA647 (Figure 1A). After mixing the two populations at a 1:1 ratio, the suspension was subjected to F/T treatment to induce fusion.

**FIGURE 1:**
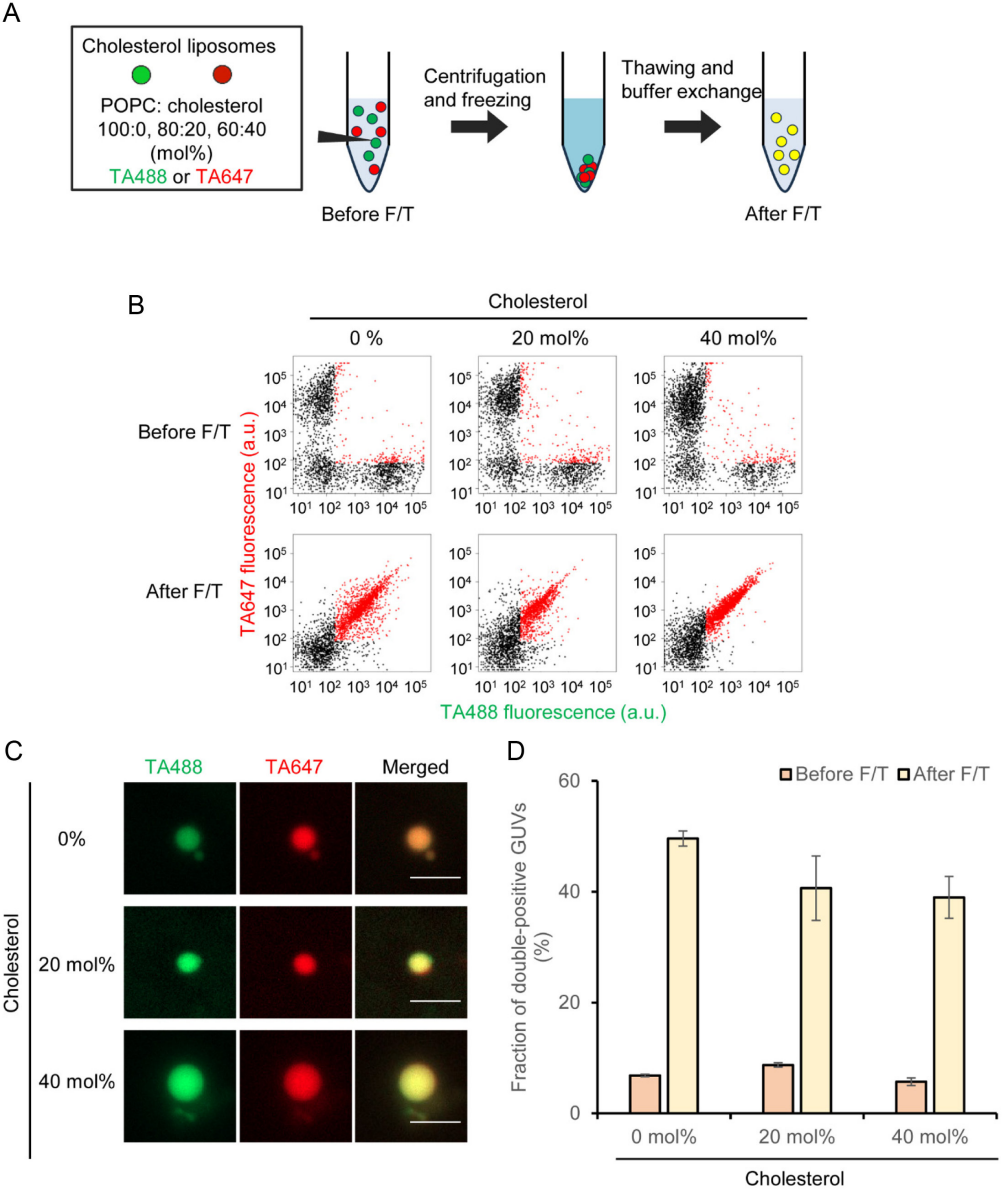
Freeze–thaw (F/T) induced fusion and content mixing of GUVs. (A) Schematic representation of the experimental procedure for F/T‐induced fusion. Two populations of GUVs encapsulating either TA488 or TA647 were mixed at equal particle numbers and subjected to rapid freezing in liquid nitrogen followed by thawing at room temperature. (B) Flow cytometry (FCM) dot plots showing TA488‐TA647 fluorescence. Vesicles exhibiting fluorescence above the threshold for both channels are indicated as red dots, corresponding to double‐positive GUVs. (C) Fluorescence microscopy images of GUVs composed of POPC with or without cholesterol, acquired after F/T treatment. TA488 (green) and TA647 (red) fluorescence signals are shown. Scale bar: 10 μm. (D) Quantification of double‐positive GUVs. Bars represent the fraction of GUVs positive for both TA488 and TA647 fluorescence, calculated from three independent experiments. Error bars show standard error of the mean (*n* = 3).

We first quantified changes in the GUV event fraction and GUV recovery after F/T (Figure S1A). Cholesterol did not affect the GUV event fraction (Figure S1B,C Before F/T). In contrast, the GUV event fraction decreased after F/T, and this decrease was more pronounced in cholesterol‐containing membranes (Figure S1B,C after F/T). Consistently, the number of GUVs also decreased after F/T across all cholesterol conditions (Figure S1D).

We next analyzed content mixing of TA488 and TA647 within individual GUVs. Consistent with previous reports [19], GUVs composed of POPC only exhibited two mutually exclusive fluorescence populations before F/T, but a substantial fraction of double‐positive GUVs emerged after F/T (Figure 1B, 0%). Fluorescence microscopy further confirmed that both fluorescent proteins were present within single GUVs as mixed luminal signals rather than membrane adsorption or liposome aggregation, demonstrating inner content mixing between GUVs (Figure 1C, 0%). Cholesterol‐containing GUVs also exhibited content mixing after F/T (Figure 1B,C, 20 and 40 mol%). However, the fraction of double‐positive GUVs was lower in cholesterol‐containing membranes compared with POPC alone (Figure 1D). This reduction depended on the presence of cholesterol but did not vary appreciably between 20 and 40 mol%.

Collectively, these results indicate that cholesterol does not affect GUV formation but slightly alters GUV behavior during F/T treatment. Because cholesterol is known to decrease membrane fluidity and lateral lipid diffusion [33, 34], the observed reduction in content mixing may reflect changes in membrane remodeling during F/T. Nevertheless, cholesterol alone exerted only a modest effect on both GUV recovery and inner content mixing during F/T, suggesting that additional membrane components may be required to substantially alter F/T‐induced fusion behavior.

### 2.2 Content Mixing Between GUVs Containing Negatively Charged Phospholipids and Cholesterol

Previous studies have reported that liposomes containing POPG exhibit reduced rupture and limited internal fluid leakage during F/T [23]. We therefore examined how the addition of cholesterol modifies the previously reported effects of POPG on GUV recovery and content mixing during F/T. GUVs encapsulating either TA488 or TA647 were prepared using membrane compositions containing 20 or 40 mol% POPG with or without 20 or 40 mol% cholesterol, mixed at a 1:1 ratio, and subjected to F/T treatment.

Across all membrane compositions, the efficiency of GUV formation prior to F/T was higher than that observed for POPC alone or POPC containing cholesterol (Figure S2A,B, before F/T). In addition, the GUV event fraction showed only minor changes before and after F/T in the POPG‐containing membranes, indicating that POPG improved GUV recovery following F/T treatment (Figure S2A,B). GUV recovery after F/T was also higher in POPG‐containing compositions than in POPC or POPC‐cholesterol membranes (Figure S2C). In membranes containing 40 mol% POPG, the forward scattering (FSC) distribution exhibited a modest shift toward lower values after F/T treatment, whereas little change was observed in membranes containing 20 mol% POPG (Figure S2D).

We next examined whether cholesterol further modified the effects observed in POPG‐containing membranes. Addition of cholesterol to POPG‐containing membranes did not substantially reduce the GUV event fraction or GUV recovery after F/T, with values comparable to or slightly higher than those observed for the corresponding POPG‐containing membranes without cholesterol (Figures S2A,B and S3A,B). In addition, GUV recovery after F/T remained higher in POPG‐containing membranes than in POPC membranes, regardless of the presence of cholesterol (Figures S2C and S3C). Thus, the improved GUV recovery during F/T was primarily associated with the presence of POPG, consistent with our previous findings, whereas cholesterol had only a limited additional effect on this property.

We next quantified content mixing between GUVs. Consistent with our previous findings on the effect of POPG during F/T [23], POPG‐containing membranes exhibited substantial fractions of double‐positive GUVs after F/T (Figures 2A,B and S3D,E). Addition of cholesterol to POPG‐containing membranes did not substantially alter the overall fraction of double‐positive GUVs compared with the corresponding POPG‐containing membranes without cholesterol (Figures 2B and S3D,E). However, the fluorescence distributions became broader in the presence of cholesterol, indicating increased heterogeneity in the extent of content mixing among individual GUVs (Figure 2A). Fluorescence microscopy confirmed the presence of GUVs containing both TA488 and TA647 after F/T (Figure 2C). In contrast, no double‐positive GUVs were identified in the fluorescence microscopy images acquired prior to F/T (Figure S4), supporting increased content mixing following F/T treatment.

**FIGURE 2:**
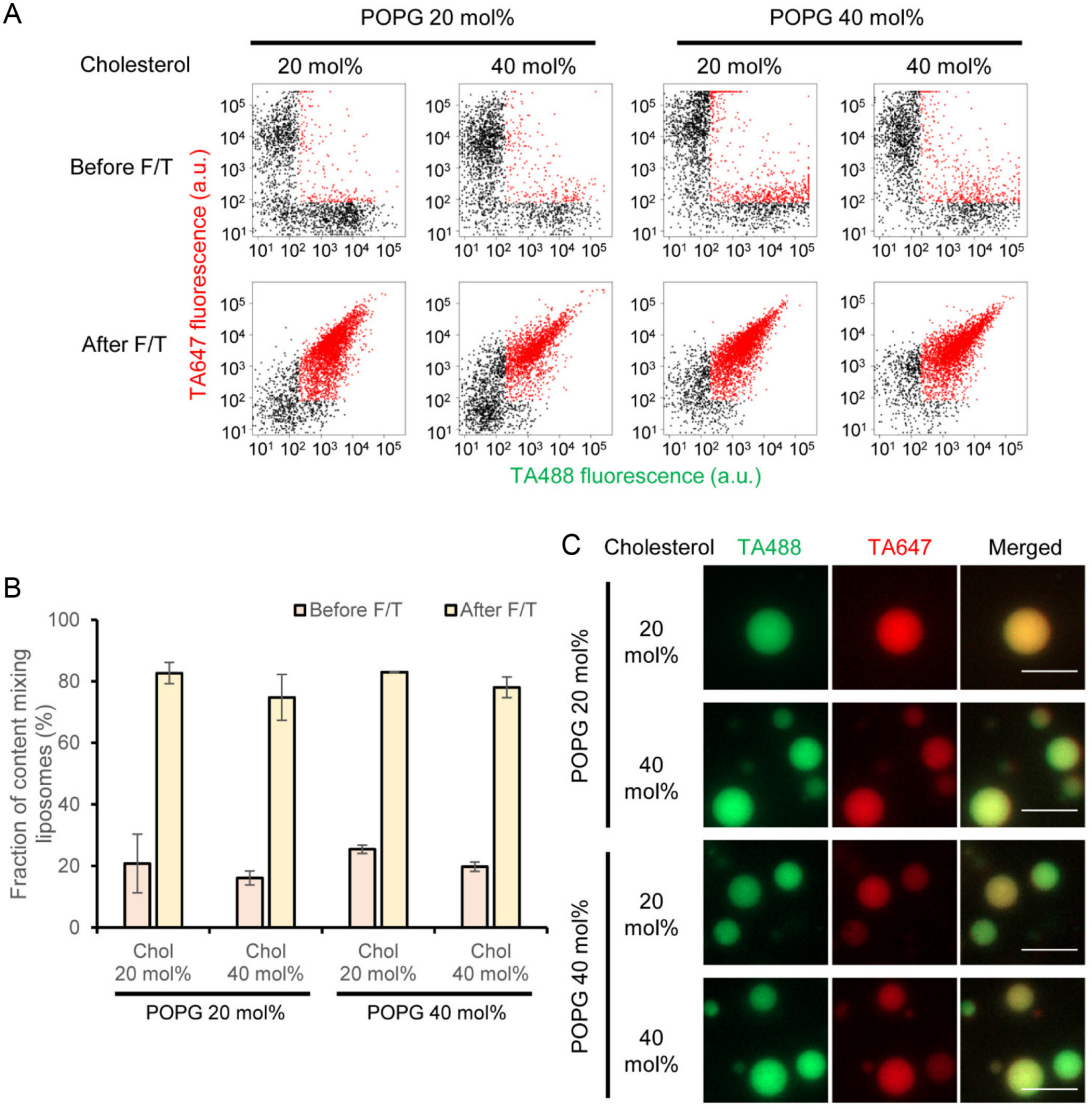
F/T induced fusion and content mixing of GUVs composed of POPG and cholesterol. (A) FCM dot plots showing TA488‐TA647 fluorescence for GUVs composed of POPC with POPG and cholesterol at the indicated molar ratios. Vesicles exhibiting fluorescence above the predefined thresholds for both channels are displayed as red dots, corresponding to double‐positve GUVs. (B) Quantification of content‐mixed GUVs. Bars represent the fraction of GUVs positive for both TA488 and TA647 fluorescence, calculated from three independent experiments. Error bars indicate the standard error of the mean (*n* = 3). (C) Fluorescence microscopy images of GUVs containing 20 or 40 mol% POPG with 20 or 40 mol% cholesterol, acquired after F/T treatment. TA488 (green) and TA647 (red) fluorescence signals are shown. Scale bar: 10 μm.

These results indicate that cholesterol modulates the pattern of content mixing in POPG‐containing membranes without substantially altering the overall fraction of mixed GUVs. In particular, the broader fluorescence distributions observed in cholesterol‐containing membranes suggest increased heterogeneity in the extent of content exchange among individual vesicles. Because cholesterol is known to increase membrane order and promote lateral heterogeneity within lipid bilayers [34], the observed changes in fluorescence distributions may reflect composition‐dependent differences in membrane behavior during F/T.

The mechanism underlying F/T‐induced content exchange may depend on the experimental conditions. In our previous liquid nitrogen F/T experiments, both content and membrane lipid mixing were observed, whereas Litschel et al. observed content exchange without detectable membrane mixing and attributed it primarily to transient membrane permeabilization during thawing [19, 20]. These differences may reflect differences in freezing protocols and thermal histories, which affect membrane mixing during F/T [19, 35]. Thus, content mixing alone does not demonstrate GUV fusion, and membrane mixing and transient permeabilization may contribute to different extents, depending on the F/T conditions. Together with the enhanced content mixing observed here, this is consistent with a model in which POPG suppresses irreversible membrane disruption while permitting reversible membrane remodeling, permeabilization, and resealing during thawing. In contrast, cholesterol did not improve GUV recovery despite its known effect on membrane order, suggesting that increased membrane order alone cannot explain the effect of POPG. These membrane remodeling processes were not directly characterized, and their molecular mechanisms remain to be determined.

### 2.3 Transcription Reaction in GUVs Containing POPG and Cholesterol

High concentrations of cholesterol (≥40 mol%) [31] or negatively charged phospholipids such as POPG (≥30% w/w) [36] have been reported to inhibit transcription reactions in liposomal systems. However, it remains unclear whether combinations of these membrane components influence transcriptional activity at concentrations that are individually noninhibitory. To address this question, GUVs encapsulating DNA, T7 RNA polymerase, and all components required for transcription were prepared using four membrane compositions, such as POPC, POPC containing 20 mol% POPG, POPC containing 20 mol% cholesterol, and POPC containing both 20 mol% POPG and 20 mol% cholesterol. The GUVs were incubated at 37 °C for 2 h, and transcriptional activity was evaluated by SYBR Green II fluorescence (Figure 3A) by using flow cytometry (FCM).

**FIGURE 3:**
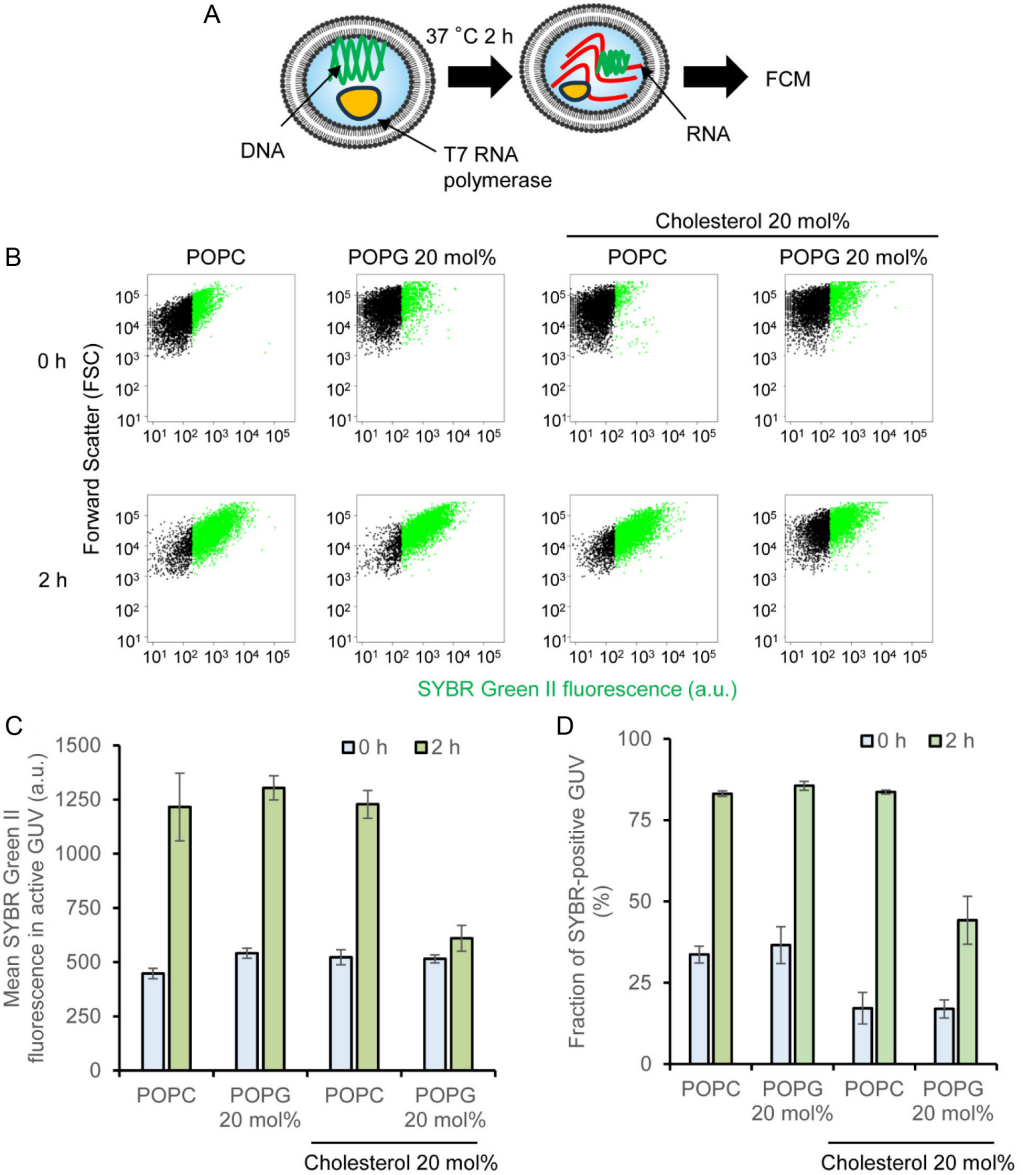
Transcription efficiency in POPC/POPG/cholesterol GUVs. (A) Schematic illustration of the transcription reaction inside GUVs. DNA and T7 RNA polymerase were coencapsulated, and RNA synthesis was monitored using SYBR Green II. (B) FCM plots showing SYBR Green II fluorescence versus FSC. Green‐gated dots indicate GUVs exhibiting fluorescence above the background level. (C) Mean SYBR Green II fluorescence intensity of SYBR Green II–positive GUVs after 2 h incubation at 37 °C. Error bars represent the standard error of the mean (*n* = 3). (D) Fraction of SYBR Green II–positive GUVs before (0 h) and after 2 h incubation, defined as the percentage of GUVs exhibiting FITC‐A fluorescence above a predefined threshold determined from the 0 h sample. Error bars represent the standard error of the mean (*n* = 3).

All membrane compositions exhibited an increase in fluorescence intensity after incubation, indicating that transcription occurred under all conditions (Figure 3B). However, comparison of the mean fluorescence intensities within the GUV gate revealed a marked reduction in RNA yield in membranes containing both POPG and cholesterol, whereas membranes containing either lipid alone exhibited transcriptional activities comparable to that of POPC membranes (Figure 3C). Two‐way analysis of variance (ANOVA) revealed significant main effects of both POPG and cholesterol, as well as a significant interaction between the two factors (*p* < 0.01), indicating that the inhibitory effects of these membrane components were dependent on their simultaneous presence. Consistently, the proportion of “SYBR‐positive GUVs,” defined as vesicles exceeding a predetermined fluorescence threshold, was substantially reduced only when both POPG and cholesterol were present (Figure 3D).

Across all membrane compositions, the GUV event fraction changed little during the 2 h incubation at 37 °C, indicating that the vesicles remained stable under the reaction conditions (Figure S5A,B). Consistent with the results shown in Figure 2, POPG‐containing membranes exhibited higher GUV event fractions than POPC or cholesterol‐containing membranes without POPG conditions (Figure S5B). These results indicate that membrane compositions that maintain high GUV stability do not necessarily support efficient transcription. In particular, the combination of POPG and cholesterol strongly suppressed RNA synthesis despite the high GUV stability associated with POPG‐containing membranes, revealing that membrane properties favorable for compartment stability are not necessarily favorable for biochemical activity.

The reduced transcriptional activity observed in GUVs containing both POPG and cholesterol is unlikely to be explained by the effects of either lipid alone because neither POPG nor cholesterol substantially affected RNA production when present individually. Instead, the marked inhibition observed only when both lipids were present suggests that membrane charge and cholesterol‐dependent membrane organization cooperatively influence the biochemical environment required for transcription. Such effects may arise from changes in membrane organization, local solute distribution, or macromolecular crowding near the membrane interface. Consistent with this interpretation, two‐way ANOVA revealed significant interaction effects between POPG and cholesterol for both the fraction of active GUVs and the fluorescence intensity of RNA‐positive GUVs, indicating that the effects of these membrane components on transcriptional activity are not simply additive. The effect of POPG on transcription observed here differed from that in our previous study [24], in which reduced RNA synthesis was observed in GUVs containing 10 or 30% (w/w) POPG. The 20 mol% POPG used in the present study corresponds to approximately 18% (w/w), suggesting that the difference cannot be explained simply by the POPG concentration. However, the transcription systems differed substantially in both DNA template length and concentration. The previous study used a 3551 bp DNA template at 100 pM, whereas the present study used an approximately 1 kb template at 1 nM. The higher template concentration and shorter template used here may increase the overall transcriptional capacity of the system and thereby reduce the apparent influence of POPG on RNA production. Because the effects of template length and concentration were not systematically examined, however, the precise origin of the different POPG dependence remains unclear. Nevertheless, the present results demonstrate that the effect of cholesterol on encapsulated transcription depends on the phospholipid composition, with marked inhibition observed specifically in combination with POPG.

### 2.4 Transcription Reaction After F/T in Liposomes With POPG and Cholesterol

The results in Figure 3 show that the combination of 20 mol% POPG and 20 mol% cholesterol markedly inhibited transcription in intact GUVs, although either membrane component alone had only a limited effect. We therefore examined whether this composition‐dependent effect was also observed when transcription was initiated following F/T‐mediated content mixing between DNA‐containing GUVs and GUVs containing transcription components. The four membrane compositions examined in Figure 3 were used to directly compare transcription before and after F/T‐mediated content mixing.

DNA liposomes encapsulating DNA and nutrient liposomes containing T7 RNA polymerase and NTPs were prepared and mixed at a 1:1 particle ratio, followed by F/T treatment (Figure 4A). The DNA concentration inside DNA liposomes was set to twice that used in the single‐GUV transcription experiments in Figure 3 to ensure sufficient substrate availability after F/T. After exchanging the external solution, samples were incubated at 37 °C for 2 h, stained with SYBR Green II, and analyzed by FCM after removing excess dye.

**FIGURE 4:**
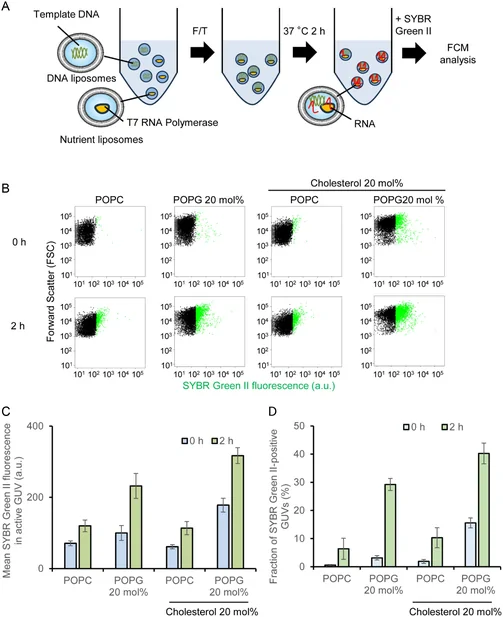
Freeze–thaw fusion delivers transcription substrates into POPC/POPG/cholesterol GUVs. (A) Schematic illustration of the F/T fusion between DNA liposomes and nutrient liposomes, followed by transcription inside the fused GUVs. (B) FCM plots showing SYBR Green II fluorescence versus FSC for GUVs after F/T. Green‐gated dots indicate GUVs exhibiting fluorescence above the background level. (C) Mean SYBR Green II fluorescence intensity of SYBR Green II–positive GUVs after 2 h incubation at 37 °C. Error bars represent the standard error of the mean (*n* = 3). (D) Fraction of SYBR Green II–positive GUVs before (0 h) and after 2 h incubation, defined as the percentage of GUVs exhibiting FITC‐A fluorescence above a predefined threshold determined from the 0 h sample. Error bars represent the standard error of the mean (*n* = 3).

Consistent with the previous study, POPG‐containing membranes exhibited a higher GUV event fraction than POPC alone or POPC containing cholesterol (Figure S6A,B). After F/T, POPC membranes containing cholesterol showed a pronounced decrease in GUV event fraction, whereas POPG‐containing membranes exhibited only a moderate decrease (Figure S6B, after F/T 0 h). Moreover, incubation at 37 °C for 2 h did not substantially alter the GUV event fraction in any membrane condition, indicating that GUV stability was largely maintained during the reaction period (Figure S6B, after F/T 2 h). FSC histograms revealed a leftward shift in the GUV population immediately after F/T, suggesting a reduction in average vesicle size. However, no further changes in the FSC distributions were observed during the subsequent 2 h of incubation, indicating that the size distribution of the remaining GUVs remained stable during the transcription reaction (Figure S6C).

Analysis of SYBR Green II fluorescence revealed increases in RNA‐associated fluorescence across all membrane compositions after F/T and 2 h of incubation (Figure 4B,C). POPC alone and POPC with cholesterol produced comparable fluorescence levels, whereas membranes containing POPG yielded higher fluorescence. Notably, membranes containing both POPG and cholesterol showed the highest mean fluorescence intensity and the largest proportion of active liposomes (Figure 4C,D).

To directly evaluate the retention of encapsulated contents during F/T, we analyzed the fluorescence intensities of TA488‐ and TA647‐containing GUVs before and after F/T (Figure S7). POPG‐containing membranes showed substantially higher fluorescence retention than that of POPC membranes. Addition of 20 mol% cholesterol to membranes containing 20 mol% POPG maintained this high level of fluorescence retention but did not result in a marked further increase compared with 20 mol% POPG alone. These results indicate that POPG contributes to the retention of encapsulated contents during F/T, whereas the additional effect of cholesterol on content retention is limited.

Taken together, the present results indicate that POPG and cholesterol affect the different processes associated with F/T in distinct and composition‐dependent ways. POPG was primarily associated with improved GUV recovery, content retention, and content mixing during F/T, consistent with its previously reported effects. In contrast, cholesterol alone did not improve these physical F/T‐associated properties. When combined with POPG, however, cholesterol substantially altered the behavior of the encapsulated transcription reaction. Notably, the combination of POPG and cholesterol strongly inhibited transcription in GUVs (Figure 3), yet it produced the highest RNA synthesis following F/T‐mediated content mixing (Figure 4). Although the high retention of encapsulated contents in POPG‐containing GUVs may contribute to enhanced transcription after F/T, retention alone does not explain the particularly high transcriptional activity of GUVs containing both POPG and cholesterol. These observations demonstrate that membrane compositions favorable for compartment stability are not necessarily optimal for encapsulated biochemical activity and that the effects of cholesterol depend strongly on the surrounding phospholipid composition. The lipid composition and compositional heterogeneity of individual GUVs after F/T were not directly determined, and F/T‐induced membrane remodeling may therefore also contribute to the observed transcriptional behavior. Further analysis of post‐F/T membrane composition will be required to resolve these mechanisms.

## 3 Conclusion

In this study, we systematically investigated the effects of cholesterol and the negatively charged phospholipid POPG on F/T‐induced liposome fusion and subsequent transcriptional reactions in artificial cells. Cholesterol alone slightly decreased both the GUV recovery rate and the efficiency of inner content mixing after F/T treatment. In contrast, POPG‐containing membranes exhibited improved GUV stability following F/T, and the combination of POPG with 20 mol% cholesterol resulted in enhanced inner content mixing efficiency.

On the other hand, the coexistence of POPG and cholesterol suppressed transcription within individual GUVs. This observation suggests that the previously reported inhibitory effects of POPG [24] and cholesterol [32] on transcription may be amplified when both membrane components are present simultaneously. However, when transcription was initiated by F/T‐induced fusion between DNA‐containing liposomes and nutrient‐containing liposomes, membranes containing both POPG and cholesterol produced the highest level of RNA synthesis. These results demonstrate that membrane composition regulates artificial cell stability, fusion efficiency, and internal biochemical activity in an interconnected rather than independent manner.

Our findings highlight the importance of membrane composition optimization for molecular delivery and reaction activation through F/T‐induced fusion in artificial cells. In particular, the combination of POPG and cholesterol provides a useful strategy for controlling fusion‐mediated artificial cell functions and offers design principles for the rational design of fusion‐based artificial cell systems.

## 4 Experimental Section

### 4.1 Materials

POPC, POPG, and cholesterol were purchased from Avanti Polar Lipids (Alabaster, AL, USA). Liquid paraffin (0.86–0.89 g/mL at 20 °C) and chloroform were purchased from Wako (Osaka, Japan). Transferrin Alexa Fluor 488 conjugate (TA488) and Transferrin Alexa Fluor 647 conjugate (TA647) were purchased from Life Technologies (Carlsbad, CA, USA). T7 RNA polymerase was purchased from Takara Bio (Shiga, Japan).

### 4.2 Liposome Preparation

GUVs were prepared using the water‐in‐oil (W/O) emulsion transfer method, as previously described [19, 24, 37]. Phospholipids (POPC, POPG) and cholesterol were dissolved in chloroform (0.1 mg/µL), mixed with liquid paraffin to obtain a 5 mg/mL lipid‐in‐oil solution, and incubated at 80 °C for 30 min to remove residual solvent. Lipids were then combined at the indicated molar ratios for each experiment.

For content‐mixing experiments, the inner aqueous solution consisted of 50 mM HEPES–KOH (pH 7.6), 350 mM potassium glutamate, 500 mM sucrose, and 1.5 µM transferrin–Alexa Fluor (TA488 or TA647, as specified). For RNA synthesis experiments, the inner solution consisted of 50 mM HEPES–KOH (pH 8.0), 350 mM potassium glutamate, 500 mM sucrose, 2 mM NTPs, 5 mM dithiothreitol (DTT), 1 ×  T7 RNA polymerase buffer, T7 RNA polymerase, and 1 nM template DNA.

The appropriate inner solution was added to the lipid–oil mixture and emulsified by vortex. The W/O emulsion was placed on ice for 10 min, layered onto the outer solution (50 mM HEPES–KOH (pH 7.6), 350 mM potassium glutamate, and 1 M glucose), and centrifuged at 18,000  ×  g for 30 min at 4 °C. The resulting liposome pellet was collected by puncturing the bottom of the tube with a needle. The external solution of the collected GUVs was exchanged with incubation buffer (50 mM HEPES–KOH (pH 7.6), 1 M glucose).

For RNA synthesis after F/T experiments, DNA liposomes were prepared using an inner solution consisting of 50 mM HEPES–KOH (pH 8.0), 350 mM potassium glutamate, 500 mM sucrose, 5 mM DTT, 1 ×  T7 RNA polymerase buffer, and 2 nM template DNA. Nutrient liposomes were prepared using an inner solution consisting of 50 mM HEPES–KOH (pH 8.0), 350 mM potassium glutamate, 500 mM sucrose, 4 mM NTPs, 5 mM DTT, 1 ×  T7 RNA polymerase buffer, and T7 RNA polymerase.

### 4.3 Freezing and Thawing of Liposome Solutions

F/T fusion of liposomes was performed as described previously [19, 20, 23], with minor modifications. Two liposome populations were mixed at equal particle numbers (1:1 particle ratio), as determined by flow cytometric particle counting, and centrifuged at 18,000  ×  g for 10 min at 4 °C. The pellet was rapidly frozen in liquid nitrogen (−196 °C) for 5 min, followed by thawing at room temperature for 10 min. After thawing, liposomes were gently resuspended and centrifuged again at 18,000 ×  g for 10 min at 4 °C. The supernatant was carefully replaced with an equal volume of fresh incubation buffer of the same composition.

### 4.4 Template DNA Preparation

Template DNA for transcription reactions was prepared by PCR amplification, as described previously [36]. Briefly, a DNA fragment encoding the T7 promoter, ribosome‐binding site, and yEGFP was amplified from a plasmid derived from pRS403 containing the yEGFP gene. The resulting PCR product was purified and used as the template for in vitro transcription reactions.

### 4.5 FCM

FCM was performed using a FACS Canto II (BD Biosciences, San Jose, CA, USA). FSC and side scatter (SSC) signals were used to identify liposomes and to define the GUV gate, as described previously [7, 38]. The gating strategy is shown in Figure S1B. All subsequent analyses were restricted to events within this GUV gate.

In fusion experiments, fluorescence from TA488 and TA647 was analyzed. TA488 was excited with a 488 nm laser, and emission was detected through a 530 ± 15 nm band‐pass filter (FITC‐A). TA647 was excited with a 633 nm HeNe laser, and emission was detected through a 660 ± 10 nm band‐pass filter (APC‐A). In transcription experiments, SYBR Green II was excited with a 488 nm laser, and emission was detected through the same 530 ± 15 nm band‐pass filter (FITC‐A).

Data analysis was performed using R (version 3.6.3), including gating, fluorescence quantification, and statistical analysis.

### 4.6 FCM Analysis

The GUV event fraction was defined as the number of events within the FSC/SSC‐based GUV gate divided by the total number of detected events. GUV recovery after F/T was calculated as the ratio of GUV event counts per unit acquisition time after F/T to those before F/T and expressed as a percentage. All measurements were performed at the same flow rate setting.

For inner content mixing assays, the fluorescence intensities of TA488 and TA647 were quantified for each vesicle. Thresholds were determined from the fluorescence distributions of GUVs containing only one fluorescent tracer. The proportion of vesicles exceeding both thresholds was defined as the double‐positive GUV ratio.

For transcription assays, transcription reaction was evaluated using the RNA‐positive fraction and the mean SYBR Green II fluorescence intensity. Mean fluorescence intensities were calculated either from all GUVs within the GUV gate or from RNA‐positive GUVs only, as indicated in the corresponding figure legends. The RNA‐positive fraction was calculated as the percentage of GUVs exhibiting SYBR Green II fluorescence above a predetermined threshold within the GUV gate. Threshold values were determined using samples prior to transcription.

### 4.7 Fluorescence Microscopy

Fluorescence images of liposomes were obtained using an Axio Observer Z1 microscope (Carl Zeiss, Germany) equipped with a 63 ×  Plan‐Neofluar oil‐immersion objective lens (NA 1.4). TA488 was excited using a 470 nm LED, and emission was detected through a 525 ± 25 nm band‐pass filter. TA647 was excited using a 590 nm LED, and emission was detected through a 629 ± 31 nm band‐pass filter. Fluorescence images were acquired and processed using ZEN software (Carl Zeiss).

### 4.8 Statistical Analysis

All experiments were independently repeated at least three times (*n* = 3), and data are presented as mean ± standard error of the mean, unless otherwise noted. Each replicate corresponded to an independently prepared liposome sample subjected to identical experimental procedures, including liposome preparation, F/T treatment, and subsequent analyses.

FCM data were acquired for at least 30,000 events per sample. No statistical methods were used to predetermine sample size. Sample sizes were selected based on common practice in the field and previous studies using similar experimental systems.

Statistical analyses were performed using R. For comparisons involving two independent variables, two‐way ANOVA was used. Differences were considered statistically significant at *p* < 0.05.

## Conflicts of Interest

The authors declare no conflicts of interest.

## Supporting information

Supplementary Material

## Data Availability

The data that support the findings of this study are available from the corresponding author upon reasonable request.
