# Supplementary material for: Effects of POPG and Cholesterol on Freeze–Thawing Mediated Content Exchange and Transcription in Giant Unilamellar Vesicles
Source: Chembiochem. 2026 Nov 5;27(21):e70594. doi: 10.1002/cbic.70594 (PMC13737446; doi:10.1002/cbic.70594)
Supplement: Supplementary file 1 [file CBIC-27-e70594-s001.pdf]

Title

Synergistic Effects of POPG and Cholesterol on Freeze-thaw Mediated Substrate Delivery into GUVs

Authors

Tsubasa Yamashina[a], Masaya Oki[a,b], and Gakushi Tsuji[a,b,*]

[a] T. Yamashina, M. Oki, G. Tsuji*

Department of Applied Chemistry and Biotechnology

Graduate School of Engineering, University of Fukui, Fukui, Japan.

3-9-1 Bunkyo, Fukui-shi, Fukui 910-8507 (Japan)

E-mail: gakushi@u-fukui.ac.jp

[b] M. Oki, G. Tsuji,

Life Science Innovation Center

University of Fukui, Fukui, Japan.

3-9-1 Bunkyo, Fukui-shi, Fukui 910-8507 (Japan)

Supplemental Figures

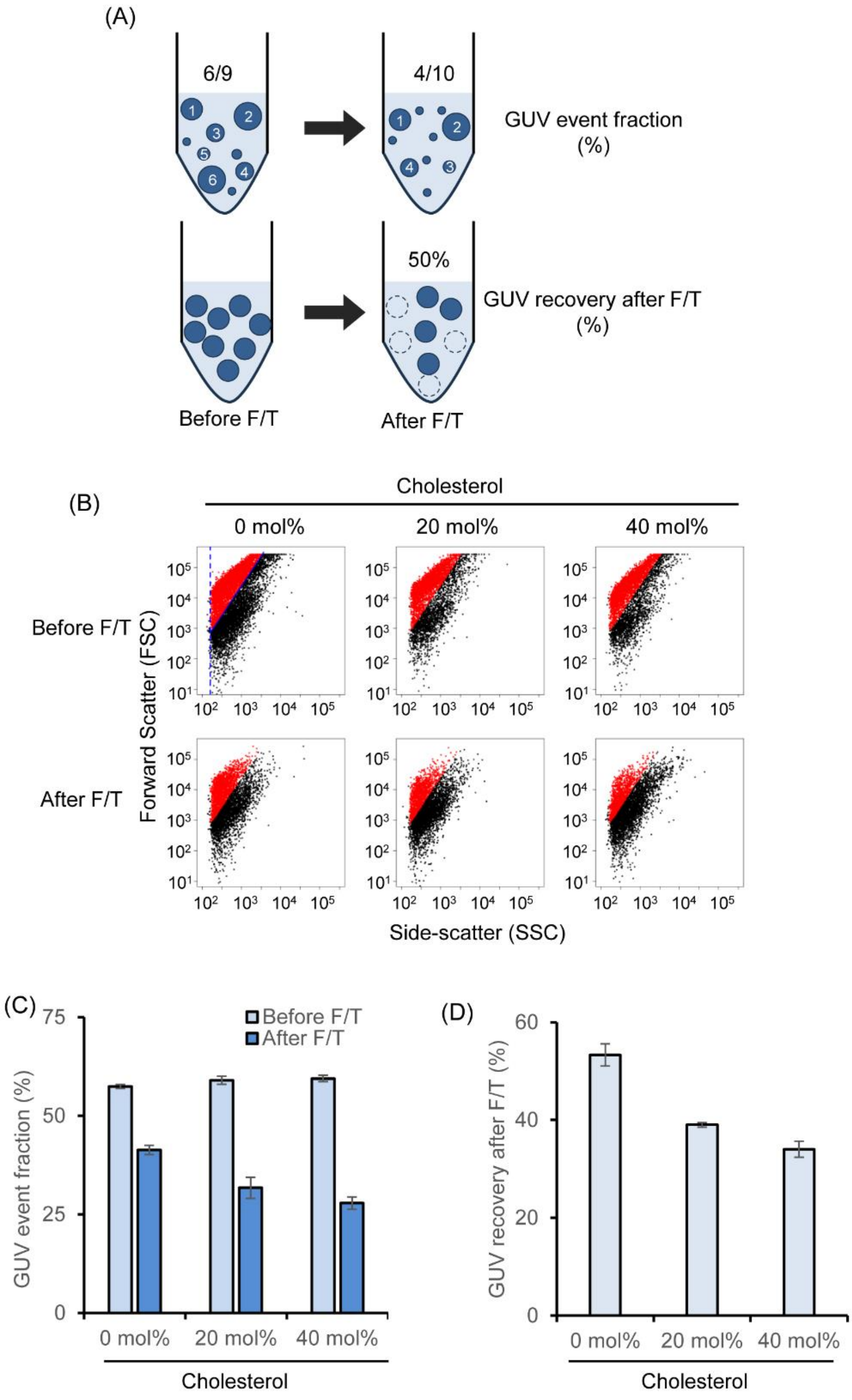

Figure S1. GUV formation efficiency and stability before and after freeze–thaw (F/T) treatment. (A) Schematic representation of the two quantitative metrics used in this study: (i) the percentage of GUVs before and after F/T, and (ii) the percentage of GUVs remaining after F/T. (B) Flow cytometry (FCM) dot plot of FSC versus SSC. Events satisfying SSC-A $\geq 10^{2.2}$ and FSC-A $\geq 0.035 \times ((\text{SSC-A})^{1.95} + 1000)$ were defined as GUVs and used for subsequent analyses. Red dots indicate vesicles within the GUV gate. (C) Average GUV event fraction under each membrane condition. Values

correspond to the proportion of red-gated events shown in (B). (D) Average percentage of GUV recovery after F/T, calculated as the ratio of GUV event counts normalized by acquisition time after and before F/T. Error bars represent the standard error of the mean (n = 3).

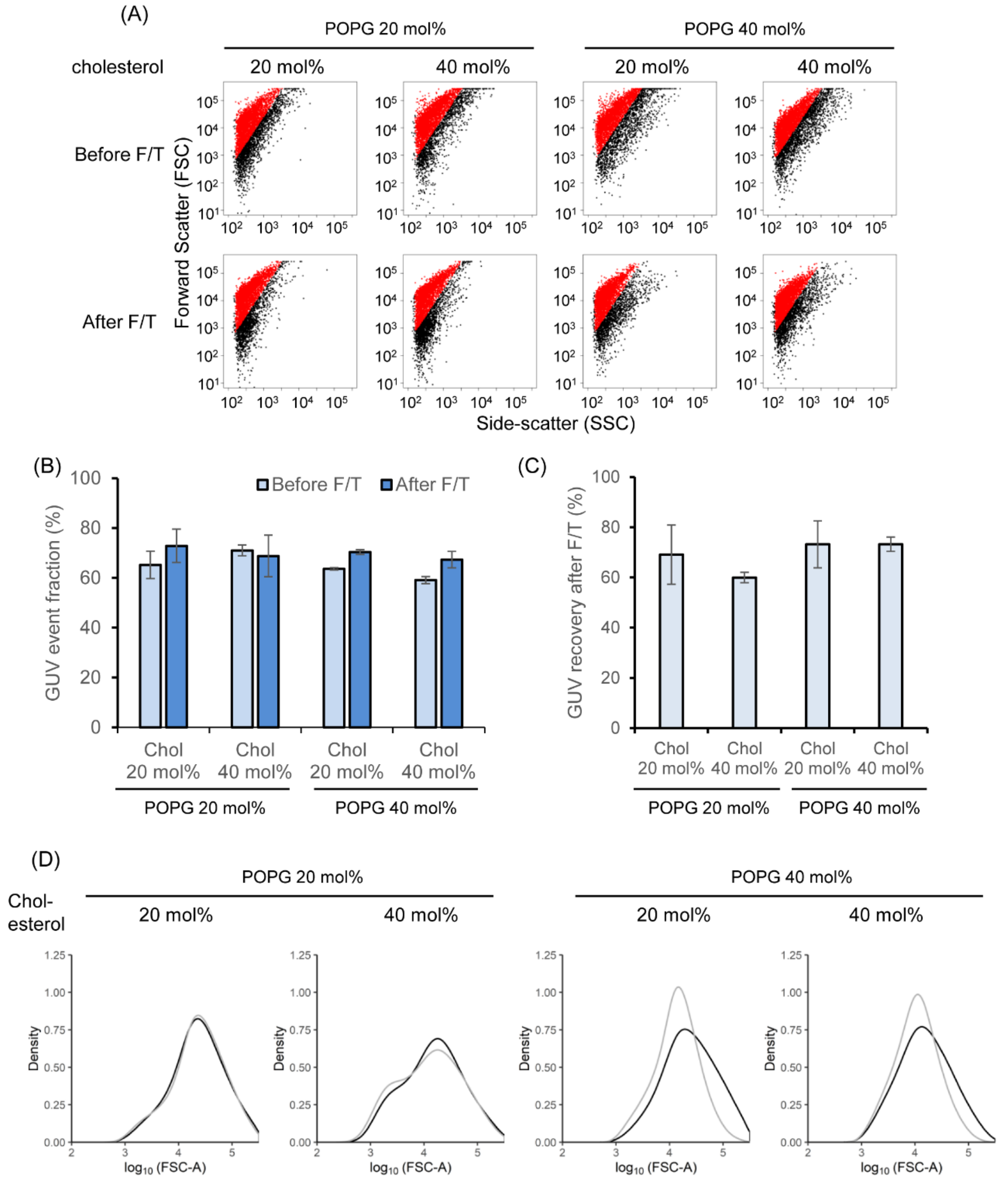

Figure S2. GUV formation efficiency and stability of POPC/POPG/cholesterol vesicles before and after F/T treatment. (A)FCM dot plots of FSC versus SSC. Red-gated dots indicate vesicles within the GUV gate. (B) Average GUV event fraction under each membrane condition. Values correspond to the proportion of red-gated particles shown in (A). Error bars represent the standard error of the mean (n = 3). (C) Average percentage of GUV recovery after F/T, calculated as the ratio of GUV event counts normalized by acquisition time after and before F/T. Error bars represent the standard error of the mean (n = 3). (D) FSC histograms of GUVs composed of POPG and cholesterol before and after F/T treatment. Histograms are shown for membrane compositions containing 20 or 40 mol% POPG combined with 20 or 40 mol% cholesterol.

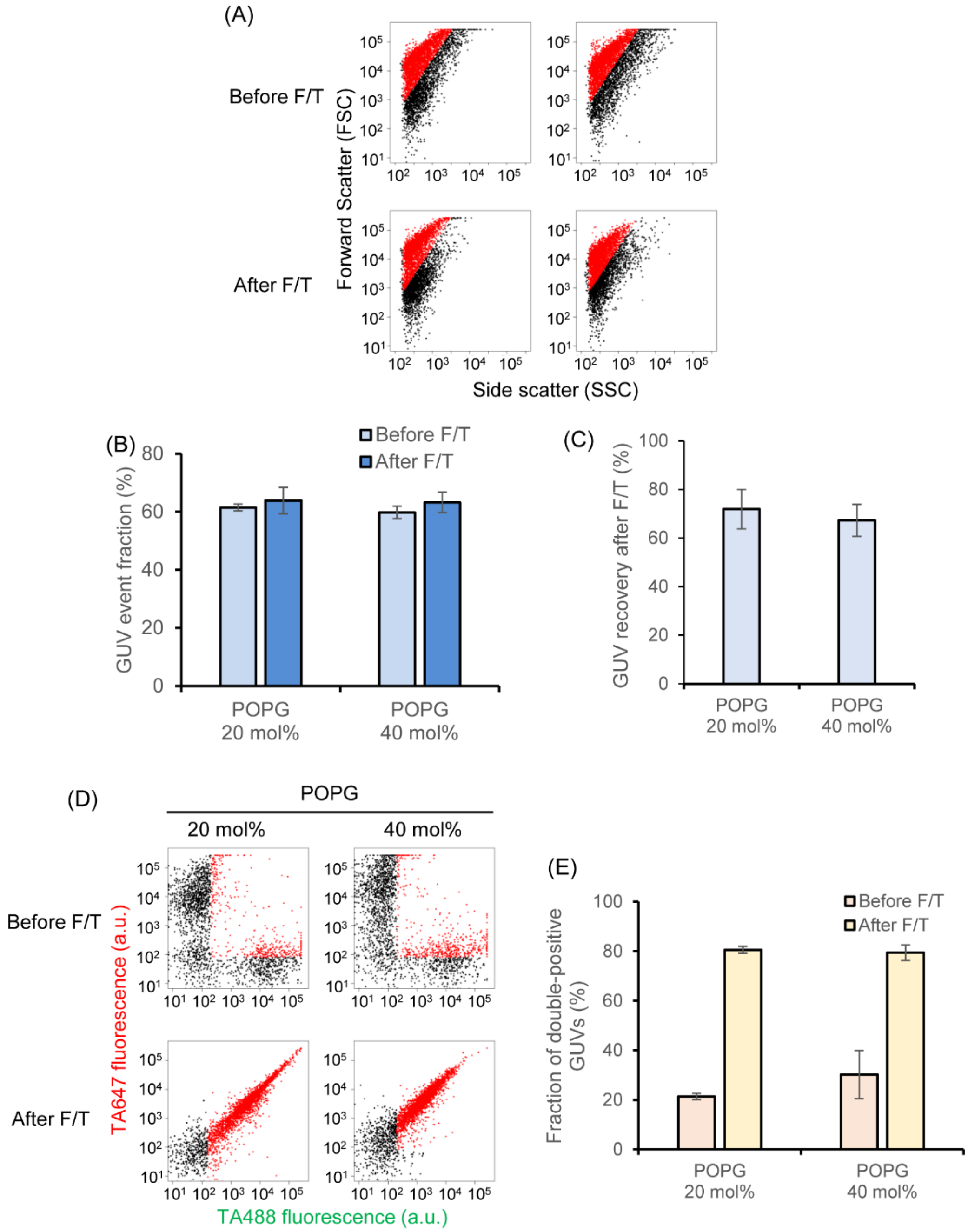

Figure S3. GUV formation, stability, and content mixing of POPC/POPG vesicles before and after F/T treatment (A) FCM dot plots of FSC versus SSC. Red-gated dots indicate vesicles within the GUV gate. (B) Average GUV event fraction under each membrane condition. Values correspond to the proportion of red-gated particles shown in (A). Error bars represent the standard error of the mean (n = 3). (C) Average percentage of GUV recovery after F/T, calculated as the ratio of GUV event counts normalized by acquisition time after and before F/T. Error bars represent the standard error of the mean (n = 3). (D) FCM dot plots showing TA488 versus TA647 fluorescence for GUVs composed of POPC with POPG at the indicated molar ratios. Vesicles exhibiting fluorescence above the predefined thresholds for both channels are displayed as red dots, corresponding to GUVs in which internal solutions were mixed after F/T treatment. (E)

Quantification of content-mixed GUVs. Bars represent the fraction of GUVs positive for both TA488 and TA647 fluorescence, calculated from three independent experiments. Error bars represent the standard error of the mean (n = 3).

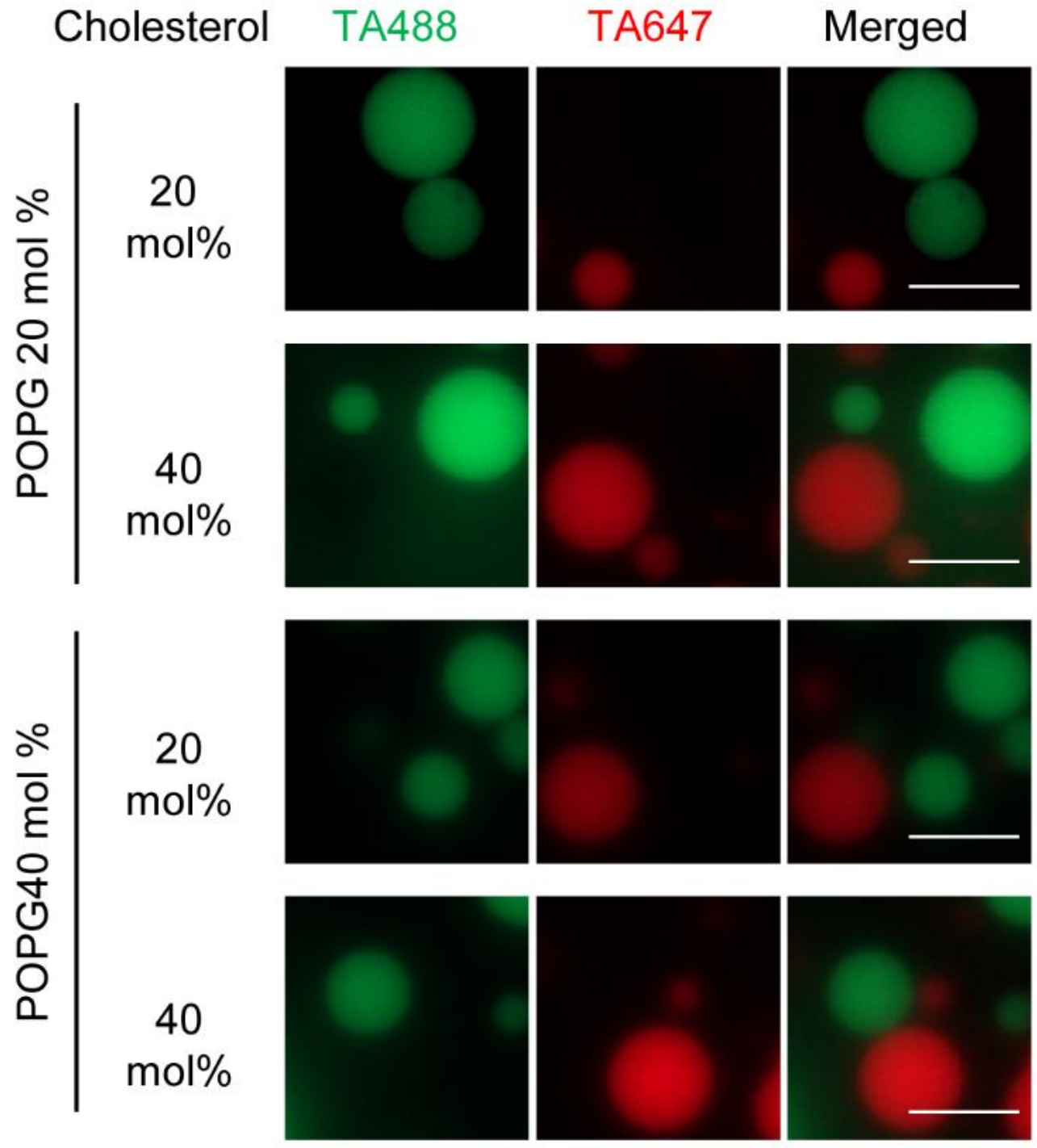

Figure S4. Fluorescence microscopy images of POPC/POPG/cholesterol GUVs before F/T treatment (A) Fluorescence microscopy images of GUVs composed of POPC with or without cholesterol before F/T treatment. TA488 (green) and TA647 (red) fluorescence signals are shown. Scale bar: 10 µm.

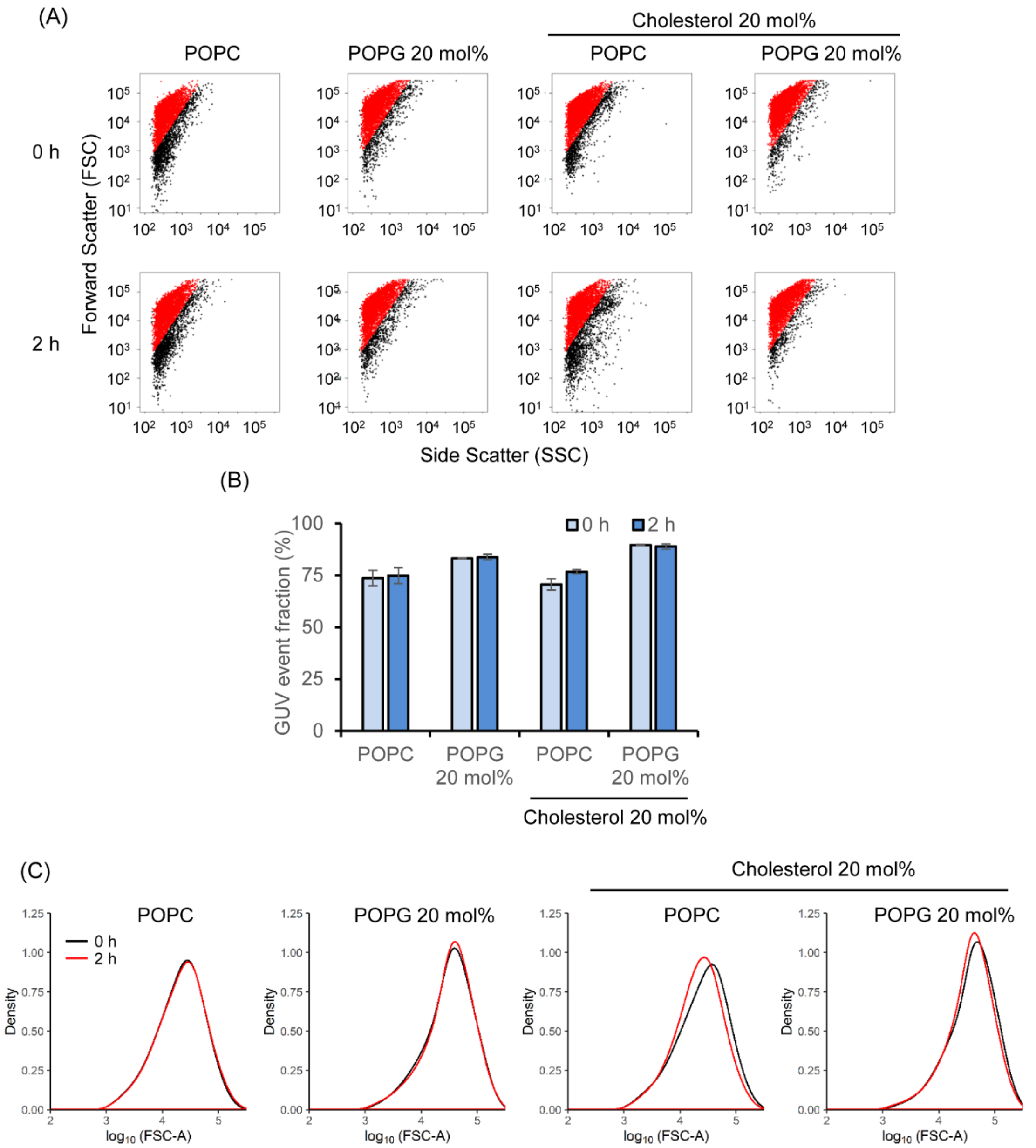

Figure S5. GUV formation efficiency and stability of POPC/POPG/cholesterol membranes during the transcription reaction (A) FCM dot plots of FSC-SSC. Red-gated dots indicate vesicles within the GUV gate. (B) Average GUV event fraction under each membrane condition. Values correspond to the proportion of red-gated particles shown in (A). Error bars represent the standard error of the mean (n = 3). (C) FSC histograms of GUVs composed of POPC, 20 mol% POPG, 20 mol% cholesterol, or 20 mol% POPG/20 mol% cholesterol before and after incubation at 37°C for 2 h.

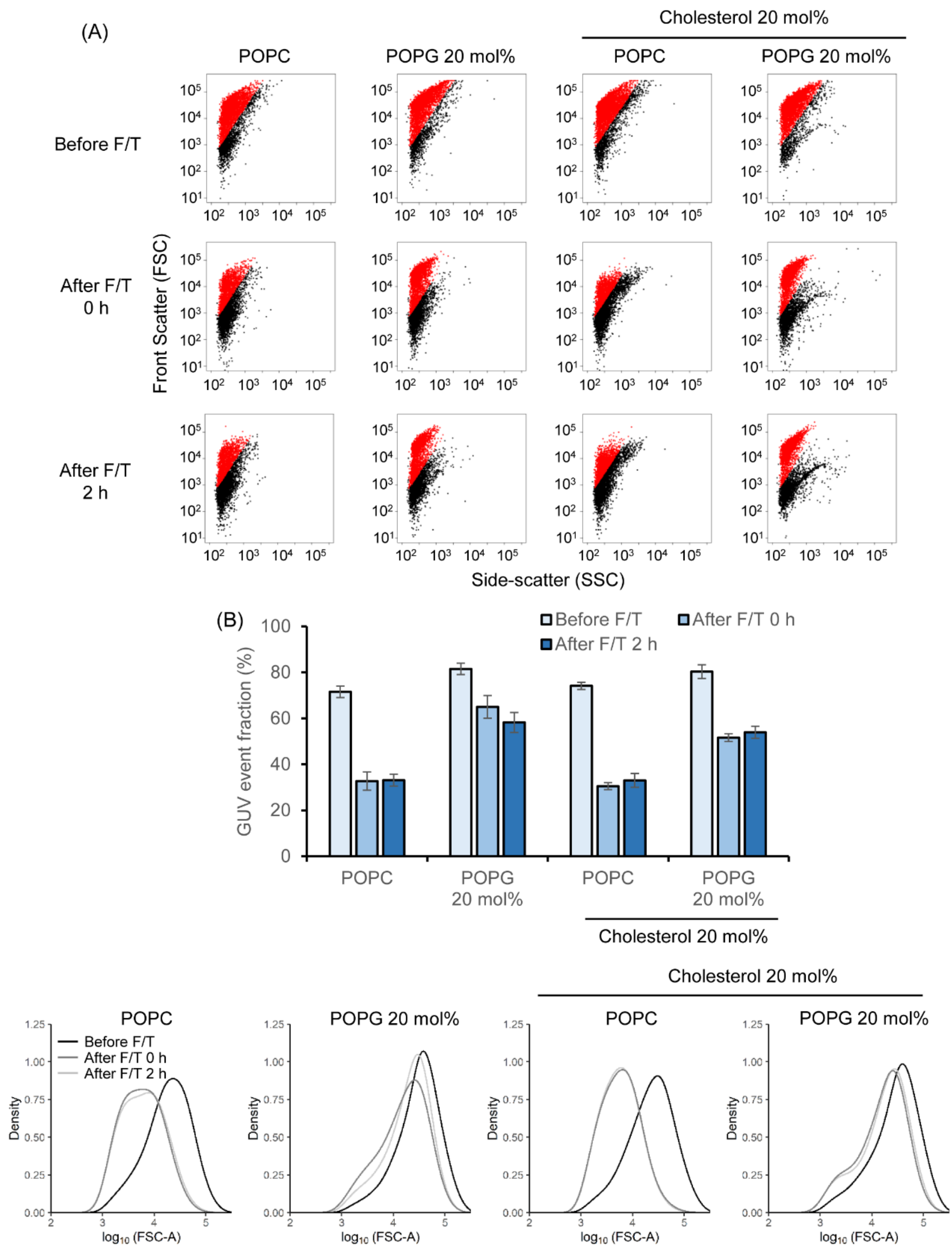

Figure S6. GUV formation efficiency and stability of POPC/POPG/cholesterol membranes during the F/T and subsequent transcription reaction (A) FCM dot plots of FSC-SSC. Red-gated dots indicate vesicles within the GUV gate. (B) Average GUV event fraction under each membrane condition. Values correspond to the proportion of red-gated particles shown in (A). Error bars represent the standard error of the mean (n = 3). (C) FSC histograms of GUVs composed of POPC, 20 mol% POPG, 20 mol% cholesterol, or 20 mol% POPG/20 mol% cholesterol before freeze–thaw (F/T) treatment, immediately after F/T (0 h), and after 2 h incubation at 37°C.

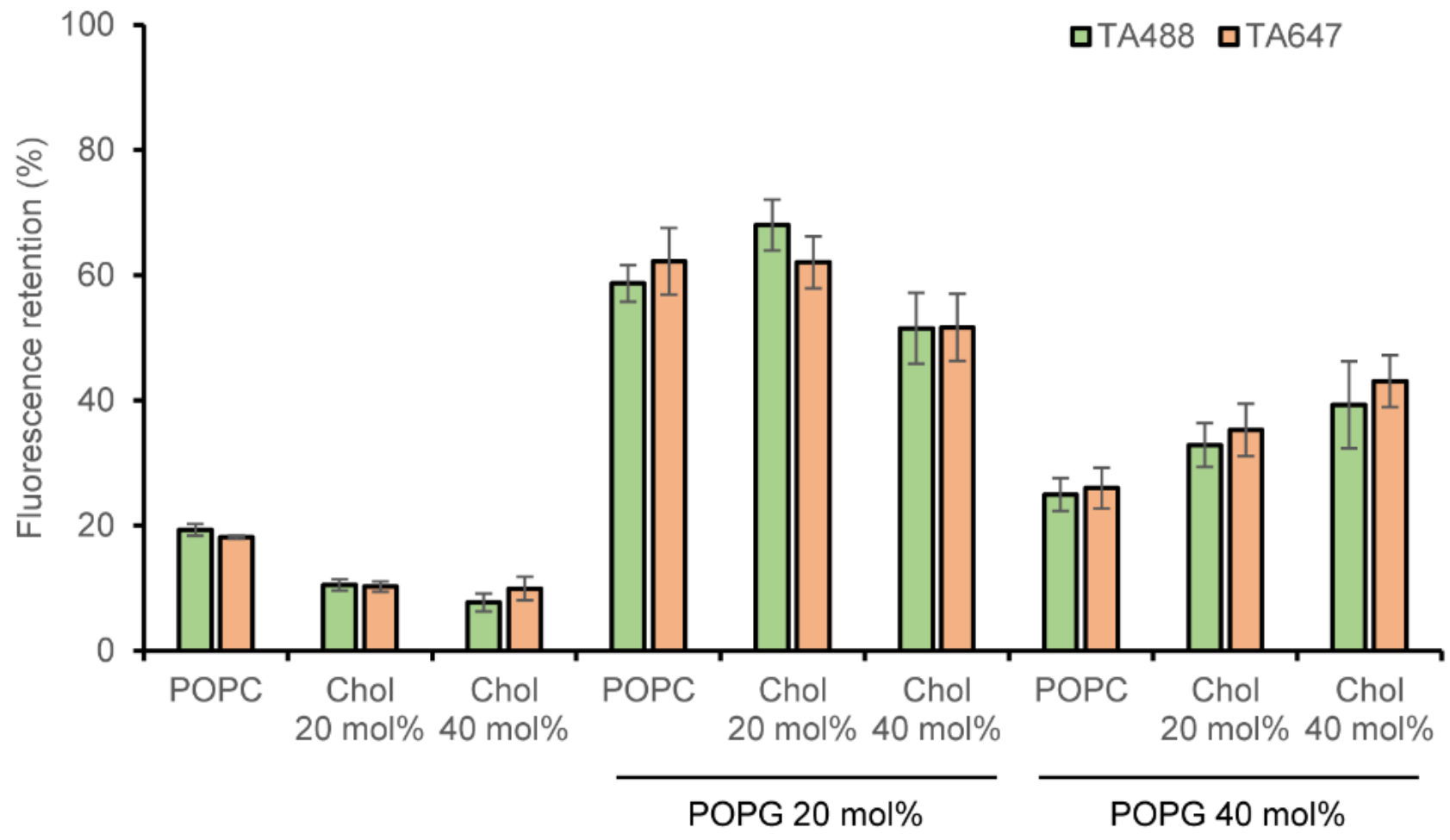

Figure S7. Fluorescence retention in POPC/POPG/cholesterol GUVs after F/T treatment. Fluorescence retention of TA488 and TA647 in GUVs with different membrane compositions after F/T treatment. Fluorescence retention was calculated as the mean fluorescence intensity of GUV-gated events after F/T treatment relative to that before F/T treatment. Error bars represent the standard error of the mean (n = 3).
